# A Prospective Study of Clinical Features of Anterior Uveitis in Taiwan

**DOI:** 10.1155/2023/9647418

**Published:** 2023-11-07

**Authors:** Wei-Yu Chiang, Shih-Chou Chen, Shwu-Jiuan Sheu, Hsi-Kung Kuo

**Affiliations:** ^1^Department of Ophthalmology, Kaohsiung Chang Gung Memorial Hospital and Chang Gung University College of Medicine, Kaohsiung City 83301, Taiwan; ^2^School of Medicine, Chang Gung University, Taoyuan City 33302, Taiwan; ^3^Department of Ophthalmology, Kaohsiung Veterans General Hospital, Kaohsiung, Taiwan; ^4^Department of Ophthalmology, Kaohsiung Medical University Hospital, Kaohsiung, Taiwan; ^5^Kaohsiung Medical University, Kaohsiung, Taiwan

## Abstract

In this study, we reported the patterns, epidemiology, and clinical features of anterior uveitis (AU) in Taiwan, an area of Eastern Asia. This prospective, cross-sectional case series study was performed to identify patients with AU at two tertiary medical centers (Kaohsiung Chang Gung Memorial Hospital and Kaohsiung Veterans General Hospital) located at the southern Taiwan between December 1, 2018, and March 31, 2020. The clinical diagnoses, ocular presentations, and laboratory data, including the results of the aqueous polymerase chain reaction tests, were investigated in these patients. A total of 112 patients, with a mean age of 48.9 years, were included. Most patients (87.5%) presented with unilateral eye disease, with 30 cases of ocular hypertension at the first presentation (27%). The most common clinical diagnoses were idiopathic AU (37.5%), human leukocyte antigen (HLA)-B27-associated acute AU (25.0%), and herpetic AU (18.8%). Among patients with herpetic AU, cytomegalovirus (CMV) was the most common pathogen (17/21, 81%). Compared to HLA-B27-associated acute AU, CMV-related AU was mostly observed in patients that were older in age, exhibited higher intraocular pressure, more keratic precipitates, greater iris atrophy, and more pseudophakia, but was least reported in those with posterior synechiae. This prospective study identified the pattern and clinical features of AU in southern Taiwan.

## 1. Introduction

Uveitis, also referred to as intraocular inflammation, which primarily involves the uveal tract with or without the involvement of the adjacent intraocular structures, is a complex inflammatory process. It is the major cause of ocular morbidity and contributes to 5–10% of visual impairment worldwide [1]. Approximately 35% of patients with uveitis experience significant vision loss and legal blindness [2]. Moreover, most affected individuals are in the working age group of 20–65 years, which causes significant socioeconomic impacts [3]. The differential diagnosis of uveitis varies widely and is influenced by several factors, such as genetic, ethnic, geographical, environmental, nutritional, and socioeconomic factors [4]. In the developing world, infections are the leading cause of uveitis, while in developed countries, idiopathic uveitis is the leading cause [5]. Therefore, determining the proper underlying etiology of this eye condition is challenging [5, 6].

Uveitis is subclassified as granulomatous or nongranulomatous anterior, intermediate, posterior, and panuveitis based on the anatomical involvement of the eye. Anterior uveitis (AU) is the most common type, with varying incidences reported in the literature worldwide [7–11]. Jakob et al. reported cases of uveitis involving the anterior, intermediate, posterior, and panuveitis in 45.5%, 22.9%, 13.5%, and 6.2% of patients, respectively, with the remaining 12.0% of cases exhibiting extrauveal manifestations (scleritis, episcleritis, keratitis, optic neuritis, myositis, and orbital inflammation) [12]. In a 22-year study from the United Kingdom, the anatomical types observed were anterior (46%), intermediate (11.1%), posterior (21.8%), and panuveitis (21.1%) [13]. Based on the Taiwan National Health Insurance Research Database, Hwang et al. conducted an epidemiology study of uveitis, in which AU was found in 77.7% of the incident cases, including 15.2%, 6.7%, and 0.4% cases of panuveitis, posterior uveitis, and intermediate uveitis, respectively [9].

AU may be benign but can often lead to severe morbidity if not treated appropriately [6, 14]. If diagnosed and treated on time, it can be resolved without long-term sequelae. Several etiologies are known to cause AU [15]. Human leukocyte antigen (HLA)-B27-associated acute AU is a distinct clinical entity that accounts for 6–13% of all AU cases in Asia [16]. Non-HLA-B27-associated AU consists of various etiologies, including Posner–Schlossman syndrome (PSS), Fuchs' heterochromic iridocyclitis (FHI), herpetic AU, juvenile idiopathic uveitis (JIA), and other panuveitis initially presenting as AU. Some patients with non-HLA-B27-associated AU may present with ocular hypertension, such as PSS, FHI, or herpetic AU. Herpetic AU caused by viruses of the Herpesviridae family, including the herpes simplex virus (HSV), varicella-zoster virus (VZV), cytomegalovirus (CMV), and Epstein–Barr virus (EBV), exhibits specific clinical features. Our previous retrospective study, which included patients with HLA-B27-negative AU with increased intraocular pressure or corneal edema, found that 41.1% of the patients were Herpesviridae-positive, with CMV being the most common etiology [17].

The pattern of AU differs across various regions of the world, and despite the scarcity of prospective studies on the etiology of AU in Eastern Asia, this study aimed to prospectively investigate the current etiologies and clinical features of AU in Taiwan.

## 2. Materials and Methods

### 2.1. Patients

This prospective study was conducted in Kaohsiung city, located in southern Taiwan, from December 2018 to March 2020. Four uveitis specialists (Dr. Shwu-Jiuan Sheu, Dr. Hsi-Kung Kuo, Dr. Shih-Chou Chen, and Dr. Wei-Yu Chiang) enrolled new patients from two tertiary medical centers (Kaohsiung Chang Gung Memorial Hospital and Kaohsiung Veterans General Hospital). The inclusion criteria were new patients with clinical manifestations of AU, diagnosed based on history, systemic symptoms, ocular examination using a slit lamp and fundoscopy, and laboratory data. In addition, image data obtained using optical coherence tomography, fundus fluorescein angiography, and B scans were determined by clinical physicians based on clinical situations. However, patients with uveitis types other than AU were excluded from the study. This study adhered to the Declaration of Helsinki protocols and was approved by the Institutional Review Board of the Chang Gung Memorial Hospital (study reference number: 201702154A3). Informed consent was obtained from all participants.

### 2.2. Protocol

The protocol for the study involved a two-step examination. The first step consisted of collecting demographics data, clinical presentations, general ocular examination, and laboratory tests, while the second step involved an aqueous humor polymerase chain reaction (PCR) test. All patients were included based on the first-step examination, with information gathered on age, sex, history of the first episode, symptom duration, acute course or not, intraocular pressure (IOP), keratic precipitates (KPs), corneal edema, hypopyon, iris atrophy, posterior synechiae (PS), and intraocular lens (IOL). An acute course was defined as a sudden onset with a limited duration of ≤3 months. The laboratory tests included a complete blood count, HLA-B27, C-reactive protein, erythrocyte sedimentation rate, antinuclear antibody, rheumatoid factor, rapid plasma reagin, *Treponema pallidum* hemagglutination, and chest and sacroiliac radiographs. If the patient had HLA-B27-associated acute AU with HLA-B27 positivity and its corresponding presentations, the PCR test could be skipped to avoid paracentesis. However, if HLA-B27-associated acute AU was not confirmed, an aqueous PCR examination was conducted to test for CMV, HSV, VZV, and EBV.

### 2.3. Aqueous Humor PCR

Under aseptic conditions and with the aid of a microscope, anterior chamber paracentesis was performed using a 27-gauge needle. Then, 0.5 ml of aqueous humor was extracted for DNA extraction and amplification. The details of the sample processing and primer information were described in our previous study [17].

### 2.4. Diagnosis Criteria

Diagnoses were based on the physicians' clinical impressions. HLA-B27-associated AU typically presents as an acute AU with symptomatic, unilateral, sudden-onset, and limited-duration anterior segment inflammation with seronegative spondyloarthropathy as a common systemic comorbidity [18, 19]. Common clinical features of herpetic AU include conjunctival congestion, corneal edema, medium-to-large mutton-fat KPs, prominent stromal edema with haze and Descemet membrane folds, anterior chamber inflammation, iris atrophy, distorted pupil, elevated IOP, laterality, and reactivation [20–23]. A diagnosis of herpetic AU was made based on typical presentations and positive aqueous PCR results. The clinical manifestations of PSS included recurrent unilateral, mild, and acute nongranulomatous AU with markedly elevated IOP, corneal edema, KPs, low-grade cell, and vague symptoms. The clinical manifestations of FHI included recurrent unilateral AU with small KPs, presence of heterochromia, a lack of synechiae, and lack of symptoms. All patients with suspected PSS and FHI underwent aqueous PCR. If PCR was positive, these cases were reclassified as herpetic AU; otherwise, they were classified as initial PSS or FHI. JIA-associated AU is diagnosed as chronic AU, and JIA is confirmed by rheumatologists [24]. Behçet's disease-associated AU is diagnosed by evidence of uveitis and concomitant oral or genital ulcers [25].

### 2.5. Statistics

In the descriptive analysis, categorical data were presented as numbers and percentages, while continuous variables were expressed as mean ± standard deviations. Comparisons between groups were carried out using the Student's *t*-test for continuous variables and the chi-square test for categorical factors. To identify significant independent predictors for differentiating HLA-B27-associated and CMV-related AU, stepwise logistic regression analysis was used in the multivariate analyses. Statistical significance was defined as a two-tailed *P* value <0.05.

## 3. Results and Discussion

A total of 112 patients (48 males and 64 females) were enrolled in this study, with ages ranging from 9 to 83 years and a mean age of 48.9 years. A majority of the patients (87.5%) presented with unilateral eye disease.

Table 1 shows the final diagnoses, which included idiopathic acute and chronic AU in 37.5% of cases, HLA-B27-associated acute AU in 25.0%, herpetic AU in 18.8%, PSS in 11.6%, FHI in 3.6%, JIA in 2.7%, and Behcet's disease in 0.9%. Among the cases of herpetic etiology, CMV was identified as the most common pathogen (17/21, 81%). Only five patients were aged <20 years (9, 12, 15, 17, and 18 years), including two with JIA-associated, two with HLA-B27-associated, and one with idiopathic AU.

The demographics and clinical manifestations of all the participants are listed in Table 2.

Thirty patients had ocular hypertension (IOP > 21 mmHg) at the first presentation (30/112, 27%). The comparison data of the ocular hypertension (IOP > 21 mmHg) and nonocular hypertension (IOP ≤ 21 mmHg) groups are also presented in Table 2. The mean IOP was 36.2 ± 9.8 vs. 12.7 ± 4.0 mmHg (*P* < 0.001), and the IOP difference from the fellow eye was 21.6 ± 10.0 vs. −1.2 ± 4.3 mmHg (*P* < 0.001). Compared to the nonocular hypertension group, the ocular hypertension group showed an older mean age (55.2 ± 10.2 vs. 46.6 ± 17.3 years, *P*=0.002), a higher proportion of males (76.7% vs. 50.0%, *P*=0.012), longer symptom duration (20.4 ± 27.8 vs. 13.1 ± 12.6 days, *P*=0.099), and a higher incidence of corneal edema (53.3% vs. 24.4%, *P*=0.004). In contrast, the ocular hypertension group had fewer cases of PS (0.0% vs. 19.5%, *P*=0.009) and HLA-B27-positive AU (10.3% vs. 33.8%, *P*=0.016) compared to the nonocular hypertension group.

In Table 3, the HLA-B27-positive (30 cases) and negative AU (79 cases) groups are compared based on various clinical features. In comparison to the HLA-B27-negative AU group, the HLA-B27-positive AU group displayed a younger mean age (41.2 ± 14.8 vs. 52.2 ± 15.7 years, *P*=0.001), a higher proportion of first episodes (60.0% vs. 34.2%, *P*=0.014), a higher incidence of acute courses (96.7% vs. 69.2%, *P*=0.002), shorter symptom duration (10.4 ± 12.5 vs. 17.9 ± 20.4 days, *P*=0.043), a lower mean IOP (12.8 ± 9.0 vs. 22.7 ± 12.7 mmHg, *P* < 0.001), a lower incidence of ocular hypertension (10.0% vs. 32.9%, *P*=0.016), a lower IOP difference from the fellow eye (−1.8 ± 8.6 vs. 8.9 ± 12.4 mmHg, *P* < 0.001), a higher frequency of corneal edema (50.0% vs. 26.6%, *P*=0.020), a higher frequency of hypopyon (16.7% vs. 1.3%, *P*=0.002), a higher frequency of iris PS (33.3% vs. 6.3%, *P* < 0.001), and a lower incidence of pseudophakias (6.7% vs. 26.6%, *P*=0.023). Among these factors, multivariate analysis identified the IOP difference from the fellow eye (*P* < 0.001), hypopyon (*P*=0.004), and corneal edema (*P*=0.007) as significant indicators that differentiate between HLA-B27-positive and HLA-B27-negative AU groups.

Table 4 compares the HLA-B27-associated acute AU group (28 cases) and with the CMV-related AU group (17 cases) based on various clinical features. The HLA-B27-associated acute AU group exhibited a younger mean age (38.8 ± 13.7 vs. 59.2 ± 9.9 years, *P* < 0.001), a higher percentage of first episodes (64.3% vs. 11.8%, *P*=0.001), a higher incidence of acute courses (96.4% vs. 58.8%, *P* = 0.003), a shorter symptom durations (9.6 ± 11.5 vs. 26.2 ± 20.4 days, *P*=0.032), a lower IOP (10.2 ± 2.6 vs. 26.3 ± 12.9 mmHg, *P* < 0.001), a lower incidence of ocular hypertension (0.0% vs. 47.1%, *P* < 0.001), a lower IOP difference from the fellow eye (−4.2 ± 3.1 vs. 11.4 ± 11.3 mmHg, *P* < 0.001), a lower frequency of corneal KPs (60.7% vs. 88.2%, *P*=0.048), a higher frequency of iris PS (35.7% vs. 0.0%, *P*=0.007), a lower frequency of iris atrophy (0.0% vs. 41.2%, *P* < 0.001), and a lower frequency of pseudophakias (7.1% vs. 35.3%, *P*=0.039), comparing to the CMV-related AU group. Multivariate analysis revealed that IOP (*P* < 0.001), IOP difference from the fellow eye (*P* < 0.001), iris atrophy (*P*=0.007), age (*P*=0.020), and pseudophakia (*P*=0.029) were significant indicators for differentiating between HLA-B27-associated and CMV-related AU groups.

## 4. Discussion

The etiologies of AU vary due to differences in geographical, environmental, and genetic factors, as well as the times [26]. Moreover, the increased availability of diagnostic testing has led to changes in the epidemiology of uveitis in recent years [27], with PCR tests being particularly useful in revising our knowledge about the etiologies of AU, especially hypertensive uveitis [20–23]. For instance, a 2004 report from Iran identified idiopathic (40.7%), HLA-B27-associated (21.5%), FHI (17.2%), JIA-associated (4.8%), and herpetic (3.8%) AU [4]. A report from Tunisia in 2007 showed idiopathic (35.5%), herpetic AU (33.7%), FHI (8.4%), HLA-associated (7.8%), and Behçet's disease-associated AU (3.0%) [28]. Another report from Turkey in 2012 assessed HLA-B27-associated (14.6%), FHI (14.6%), herpetic (13.3%), and Behçet's disease-associated AU (6.6%) [8]. In 2017, a 10-year retrospective analysis from Taiwan showed HLA-B27-associated (38.8%), idiopathic (25.4%), PSS (10.9%), and herpetic AU (10.1%) [7]. Our study not only identified idiopathic and HLA-B27-associated acute AU but also found a higher percentage of herpetic AU than previously reported. This emphasizes the crucial role of modern PCR tests in the etiology diagnosis of AU.

HLA-B27 is the most well-known immune biomarker for AU. The seropositivity rate of HLA-B27 varies among different ethnicities, with a reported rate of 7.7% in a healthy Taiwanese population [29]. HLA-B27-associated acute AU has distinct characteristics compared to other forms of AU. Moreover, 50–75% of patients with HLA-B27-associated acute AU have seronegative spondyloarthropathy, with ankylosing spondylitis (AS) being the most common diagnosis [18]. A Chinese report indicated that the prevalence of acute AU in patients with AS was 15.8%, which may be associated with high disease activity, poor functional ability, and advanced physical impairment [30]. Conversely, acute AU is the most common ocular manifestation in HLA-B27-positive seronegative spondyloarthropathies [31]. HLA-B27-associated AU classically presents as an acute AU with symptomatic, unilateral, sudden-onset, and limited-duration anterior segment inflammation [19]. In most cases, the first attack of HLA-B27-associated acute AU occurs between 20 and 40 years of age, which is about 10 years younger than that observed in patients with HLA-B27-negative AU [18]. Male preponderance was observed, with men being affected 1.5–2.5 times more frequently than females [32, 33]. In our study, 28 patients with HLA-B27-associated acute AU exhibited a mean age of 38.8 years, an acute course of 96.4%, a mean symptom duration of only 9.6 days, but only 46.4% of occurrences in males (Table 3).

Ocular hypertension is a specific sign of active uveitis, especially in non-HLA-B27-associated AU. The increased availability of PCR testing has improved the diagnosis of hypertensive AU by detecting herpetic etiologies and associating herpetic AU with previously presumed PSS or FHI. For instance, both CMV and the Rubella virus have been implicated as etiologies of PSS or FHI [34–37]. In the current study, ocular hypertension accounted for 26.8% of all cases (30/112, Table 2). About 30.0% (9/30) of ocular hypertension cases had positive PCR test results, and all were CMV-related. A study in Thailand defined ocular hypertension as IOP >25 mmHg and found 32% PCR positivity for Herpesviridae, including 19% for CMV, 10% for HSV, and 3% for VZV [38]. In our study, 27 cases had IOP >25 mmHg and 25.9% (7/27) were Herpesviridae PCR-positive, with all being CMV-related. Our previous retrospective review and this prospective study both confirmed a high percentage of CMV-related AU [17]. CMV seroprevalence was found to be highest in South America, Africa, and Asia and lowest in Western Europe and the United States [39]. This may reflect the high CMV carrier rate in Asia. The characteristics of CMV-related AU were reported in a previous study, with the mean age of 55.5 years, a mean IOP of 29.2 mmHg, KP in 91.4% of cases, and iris atrophy in 25.7% of cases [40]. Our study revealed similar characteristics, with a mean age of 59.2 years, the mean IOP of 26.2 mmHg, KP in 88.2% of cases, and iris atrophy in 41.2% of cases (Table 4).

Apart from idiopathic AU, the two most prevalent specific diseases in our study were HLA-B27-associated acute and CMV-related AU. CMV-related AU is highly prevalent in Asia and typically presents with mild anterior chamber inflammation and elevated IOP. [41] We compared the characteristics of HLA-B27-associated acute AU and CMV-related AU and summarized the differences in Table 4. HLA-B27-associated acute AU would be the more likely etiology than CMV-related AU if patients with AU present at a younger age, have a lower IOP difference from the fellow eye, experience their first episode, have an acute course, a shorter symptom duration, more PS, and less iris atrophy. For patients with AU and ocular hypertension, PCR analysis of the anterior chamber fluid is essential to detecting herpetic etiology. The CMV-related AU group showed older age, more iris atrophy, and more pseudophakia than the non-CMV-related AU group (Table 4).

This prospective study has some limitations. Firstly, the number of patients included was small. Secondly, some patients may have received initial treatment at local clinics or other facilities prior to transferring to our tertiary medical hospital, which may have affected the ocular presentation. Details of treatment, such as the use of corticosteroids, IOP-controlling agents, and mydriatics, were not recorded. This may have led to steroid-induced ocular hypertension and reduced the number of cases due to IOP-lowering medications. In addition, we did not use a laser flare meter to measure the degree of anterior chamber inflammation. Third, this study only enrolled patients from tertiary referral centers, which may not represent the overall epidemiology of this area. However, this study provides clinicians with guidelines for managing patients with AU. First, the protocol mentioned in the Methods section can be implanted in clinical practice. Among the several clinical parameters, IOP is a strong indicator for differential diagnoses. Second, PCR is a useful tool for identifying herpetic etiology, particularly in cases of ocular hypertension.

## 5. Conclusions

This prospective study identified the epidemiological and clinical features of AU in southern Taiwan. The most common etiologies of AU were idiopathic acute, HLA-B27-associated, and CMV-related AU. PCR testing is an essential adjunct in the diagnosis of AU, especially for AU with ocular hypertension. This study demonstrated further information about subgroups of AU, such as ocular hypertension, HLA-B27, and CMV-related AU groups. AU with ocular hypertension may present in older age, more males, longer symptom duration, more corneal edema, less iris PS, and less HLA-B27-positive. The significant indicators for distinguishing between HLA-B27-positive and negative AU groups included the IOP difference from the fellow eye, hypopyon, and corneal edema. The significant indicators for distinguishing between HLA-B27-associated and CMV-related AU groups included IOP, IOP difference from the fellow eye, iris atrophy, age, and pseudophakia. These characteristics can provide ophthalmologists some clues for the differential diagnosis of AU in clinical practice.

## Figures and Tables

**Table 1:** The clinical diagnosis of anterior uveitis.

Idiopathic AU (acute and chronic)	37.5%, 42/112
HLA-B27-associated acute AU	25.0%, 28/112
Herpetic AU (CMV, HSV, VZV, EBV)	18.8%, 21/112
Posner–Schlossman syndrome (PSS)	11.6%, 13/112
Fuchs heterochromic iridocyclitis (FHI)	3.6%, 4/112
Juvenile idiopathic uveitis (JIA)	2.7%, 3/112
Behcet's disease	0.9%, 1/112

AU, anterior uveitis; HLA, human leukocyte antigen; CMV, cytomegalovirus; HSV, herpes simplex virus; VZV, varicella-zoster virus; EBV, Epstein–Barr virus.

**Table 2:** The comparison between ocular hypertension (IOP > 21) and nonocular hypertension (IOP ≦ 21).

	Total	IOP > 21 mmHg	IOP ≦ 21 mmHg	*P* value
Case no.	112	30	82	
IOP (mmHg)	19.9 ± 12.6 (6.6–55.7)	36.2 ± 9.8	12.7 ± 4.0	<0.001^*∗*^
IOP difference from fellow eye (mmHg)	5.8 ± 12.5 (−11.2-42.4)	21.6 ± 10.0	−1.2 ± 4.3	<0.001^*∗*^
Age (years)	48.9 ± 16.1 (9–83)	55.2 ± 10.2	46.6 ± 17.3	0.002^*∗*^
Male	48 (42.9%)	76.7%	50.0%	0.012^*∗*^
First episode	46 (41.1%)	30.0%	45.1%	0.150
Acute course	86 (77.5%)	73.3%	79.0%	0.525
Symptom duration (days)	15.0 ± 18.0 (1–120)	20.4 ± 27.8	13.1 ± 12.6	0.099
OU	14 (12.5%)	3.3%	15.9%	0.076
KP	81 (72.3%)	83.3%	68.3%	0.115
K edema	36 (32.1%)	53.3%	24.4%	0.004^*∗*^
Hypopyon	6 (5.4%)	0.0%	7.3%	0.128
PS	16 (14.3%)	0.0%	19.5%	0.009^*∗*^
Iris atrophy	14 (12.5%)	20.0%	9.8%	0.147
Pseudophakia	23 (20.5%)	20.7%	20.0%	0.932
HLA-B27	30 (27.5%)	10.3%	33.8%	0.016^*∗*^
PCR(+)	21/61 (34.4%)	9/30 (30.0%)	12/82 (14.6%)	0.065
CMV	17/21	9	8	0.311
HSV	1/21	0	1	0.385
VZV	1/21	0	1	0.367
EBV	2/21	0	2	0.182

IOP, intraocular pressure; OU, both eyes; K, cornea; KP, keratic precipitates; PS, posterior synechiae; PCR, polymerase chain reaction; CMV, cytomegalovirus; HSV, herpes simplex virus; VZV, varicella-zoster virus; EBV, Epstein–Barr virus.

**Table 3:** The comparison between AU with HLA-B27(+) and HLA-B27(−).

	HLA-B27(+)	HLA-B27(−)	*P* value
Case no.	30	79	
IOP	12.8 ± 9.0	22.7 ± 12.7	<0.001^*∗*^
IOP > 21	10.0%	32.9%	0.016^*∗*^
IOP difference from fellow eye (mmHg)	−1.8 ± 8.6	8.9 ± 12.4	<0.001^*∗*^
Age (years)	41.2 ± 14.8	52.2 ± 15.7	0.001^*∗*^
Male	46.7%	60.8%	0.185
First episode	60.0%	34.2%	0.014^*∗*^
Acute course	96.7%	69.2%	0.002^*∗*^
Symptom duration (day)	10.4 ± 12.5	17.9 ± 20.4	0.043^*∗*^
OU	3.3%	15.2%	0.088
KP	66.7%	77.2%	0.260
K edema	50.0%	26.6%	0.020^*∗*^
Hypopyon	16.7%	1.3%	0.002^*∗*^
PS	33.3%	6.3%	<0.001^*∗*^
Iris atrophy	3.3%	16.5%	0.067
Pseudophakia	6.7%	26.6%	0.023^*∗*^

HLA, human leukocyte antigen; IOP, intraocular pressure; OU, both eyes; K, cornea; KP, keratic precipitates; PS, posterior synechiae; PCR, polymerase chain reaction; CMV, cytomegalovirus.

**Table 4:** The comparison between HLA-B27-associated acute AU and CMV AU.

	HLA-B27	CMV	*P* value
Case no.	28	17	
IOP	10.2 ± 2.6	26.3 ± 12.9	<0.001^*∗*^
IOP > 21	0.0%	47.1%	<0.001^*∗*^
IOP difference from fellow eye (mmHg)	−4.2 ± 3.1	11.4 ± 11.3	<0.001^*∗*^
Age (years)	38.8 ± 13.7	59.2 ± 9.9	<0.001^*∗*^
Male	46.4%	70.6%	0.114
First episode	64.3%	11.8%	0.001^*∗*^
Acute course	96.4%	58.8%	0.003^*∗*^
Symptom duration (days)	9.6 ± 11.5	26.2 ± 20.4	0.032^*∗*^
OU	3.6%	0.0%	1.000
KP	60.7%	88.2%	0.048^*∗*^
K edema	42.9%	29.4%	0.367
Hypopyon	17.9%	0.0%	0.140
PS	35.7%	0.0%	0.007^*∗*^
Iris atrophy	0.0%	41.2%	<0.001^*∗*^
Pseudophakia	7.1%	35.3%	0.039^*∗*^

HLA, human leukocyte antigen; IOP, intraocular pressure; OU, both eyes; K, cornea; KP, keratic precipitates; PS, posterior synechiae; PCR, polymerase chain reaction; CMV, cytomegalovirus.

## Data Availability

The data used to support the findings of this study are available from the corresponding author upon request.
